# Iatrogenic tracheal laceration due to rigid bronchoscopy treated by endoscopic stent placement: a case report

**DOI:** 10.1093/jscr/rjad356

**Published:** 2023-10-15

**Authors:** Nozomu Motono, Takaki Mizoguchi, Mashahito Ishikawa, Shun Iwai, Yoshihito Iijima, Hidetaka Uramoto

**Affiliations:** Department of Thoracic Surgery, Kanazawa Medical University, Uchinada, Ishikawa, Japan; Department of Thoracic Surgery, Kanazawa Medical University, Uchinada, Ishikawa, Japan; Department of Thoracic Surgery, Kanazawa Medical University, Uchinada, Ishikawa, Japan; Department of Thoracic Surgery, Kanazawa Medical University, Uchinada, Ishikawa, Japan; Department of Thoracic Surgery, Kanazawa Medical University, Uchinada, Ishikawa, Japan; Department of Thoracic Surgery, Kanazawa Medical University, Uchinada, Ishikawa, Japan

## Abstract

Although rigid bronchoscopy may lead to tracheal injury, the incidence is unknown. A 59-year-old woman diagnosed with clinical stage IV esophageal cancer was scheduled to undergo placement of a silicon Y-stent by rigid bronchoscopy to address tracheal stenosis. When the tumor was cored out by rigid bronchoscopy, perforation of the lower trachea occurred, and a silicon Y-stent was inserted to cover the tracheal fistula. Chest X-ray revealed right pneumothorax, and chest drainage was performed. When spontaneous ventilation was confirmed, the patient was weaned from the ventilator in the operating room. Chest computed tomography immediately after surgery showed an air space on the right side of the stent. The space gradually disappeared over time, and no air leakage was observed. The chest drain was removed on postoperative Day 12. Conservative treatment using a silicon Y-stent for iatrogenic tracheal injury due to rigid bronchoscopy is safe.

## INTRODUCTION

Iatrogenic tracheal injury (ITI) is a rare but life-threatening complication. The most common cause of ITI is intubation with an endotracheal tube [1, 2]. Although some cases have required urgent thoracotomy, cases have also been reported in which spontaneous healing of the tracheal injury has occurred with conservative management [3–10]. Although rigid bronchoscopy has been used and is preferred in the field of interventional bronchoscopy, rigid bronchoscopy is a potentially hazardous technique [11, 12].

We report the case of a patient who was treated by deployment of a silicone Y-stent for tracheal perforation associated with rigid bronchoscopy.

## CASE REPORT

**Figure 1:**
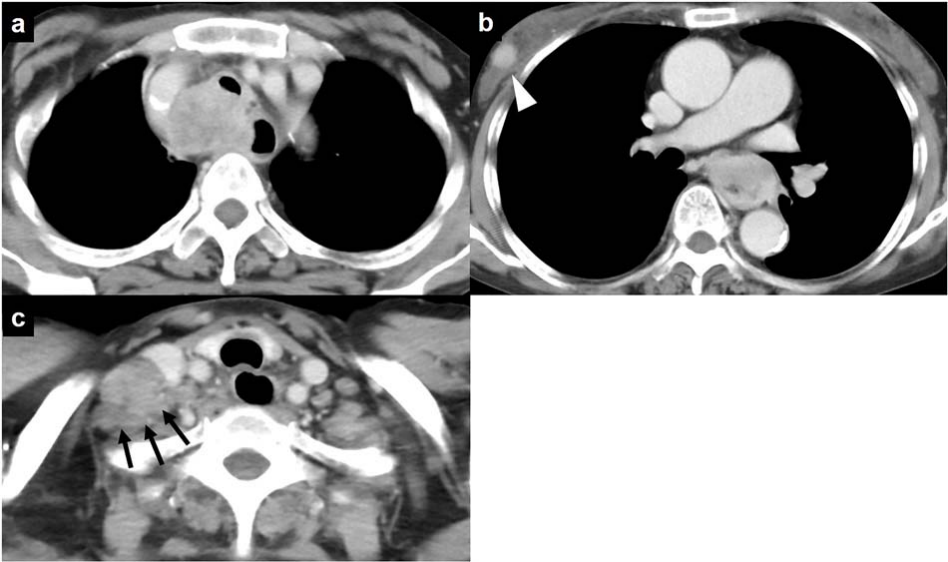
Chest computed tomography of a 59-year-old woman who was admitted to our hospital; **(a)** a mass is visible invading the trachea in the upper mediastinum; **(b)** an esophageal mass and a mass in the subcutaneous tissue of the right chest wall (arrowhead) are visible; **(c)** a mass in the right cervical region is also present (black arrows).

**Figure 2:**
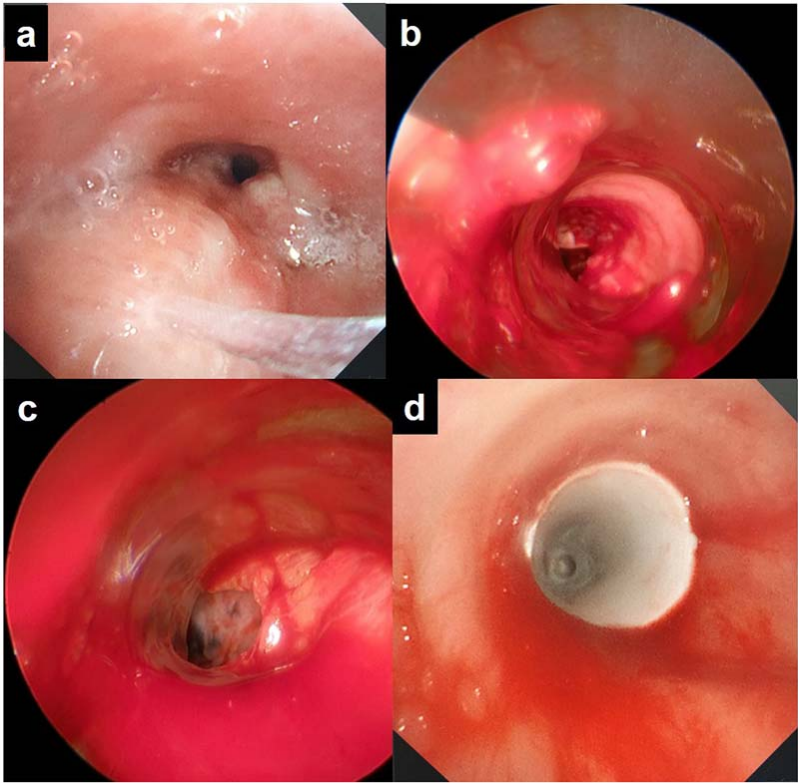
Rigid bronchoscopic findings: **(a)** the trachea is narrowed and has been invaded by the upper mediastinal mass; **(b)** perforation of the lower trachea is visible; **(c)** lung tissue is visible from the trachea; **(d)** a silicon Y-stent was inserted to cover the tracheal fistula.

**Figure 3:**
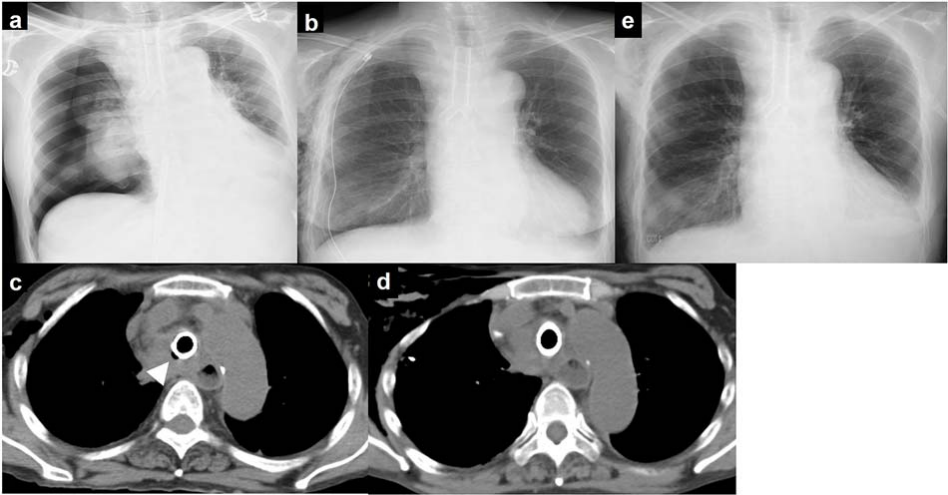
**(a)** chest X-ray showing right pneumothorax; **(b)** chest drainage was performed; **(c)** chest computed tomography immediately after surgery showing an air space on the right side of the stent; **(d)** the air space on the right side of the stent gradually disappeared; **(e)** chest X-ray showing no recurrence of pneumothorax.

A 59-year-old woman presented in respiratory distress. We identified a mass invading the trachea in the upper mediastinum, an esophageal mass, a mass in the subcutaneous tissue of the right chest wall and a mass in the right cervical area by computed tomography (Fig. 1a–c). Fine-needle aspiration for the right cervical mass was performed, and a diagnosis of squamous cell carcinoma was obtained. The patient was diagnosed as having clinical stage IV esophageal cancer. Because of respiratory distress due to airway stenosis, we planned to place a silicon Y-stent (Dumon Y; outer diameter of the main part, 14 mm; outer diameter of the branching part, 10 mm; Novatech SA, La Ciotat Cedex, France) by rigid bronchoscopy prior to chemotherapy. Rigid bronchoscopy was performed under general anesthesia combined with extracorporeal membrane oxygenation and revealed stenosis of the trachea (Fig. 2a). When the tumor was cored out by rigid bronchoscopy, perforation of the lower trachea occurred (Fig. 2b and c). A silicon Y-stent was then inserted to cover the tracheal fistula (Fig. 2d). Chest X-ray revealed right pneumothorax, and chest drainage was performed (Fig. 3a and b). The patient was weaned from extracorporeal membrane oxygenation when spontaneous ventilation was maintained; this was done in the operating room.

Chest computed tomography immediately after surgery showed an air space on the right side of the stent (Fig. 3c). The space gradually disappeared over time, and no air leakage was observed (Fig. 3d). The chest drain was removed on postoperative Day 12, and there was no recurrence of pneumothorax (Fig. 3e).

## DISCUSSION

ITI is rare; however, the most common reported cause is intubation with an endotracheal tube [1, 2]. Although there are no prospective studies reporting the incidence of ITI due to endotracheal tube intubation, the reported incidence is 0.05–0.37% [2]. Although rigid bronchoscopy may result in tracheal injury, the incidence is unknown [11, 12]. In this case, ITI occurred during rigid bronchoscopy and was relieved by stent placement. To our knowledge, there are no previous similar reports, and we judged this case to be beneficial.

There are some reports that the size (<5 cm) or depth of the tracheal laceration is important in determining the treatment strategy [4, 5, 8, 9]. However, there are also reports that even relatively large lacerations (up to 9 cm) can heal with conservative therapy [7, 10, 13]. Furthermore, there is a report describing an ITI in the lower trachea that was treated with a silicon Y-stent [10]. Although the size of the laceration in the current case was unknown, the laceration size was estimated at 3 cm. The location of the laceration was the lower trachea by rigid bronchoscopy, which was considered an indication for the placement of a silicon Y-stent.

The criteria for surgical intervention for tracheal ITI have been reported [14]. In accordance with these criteria, surgical intervention is recommended for insufficient mechanical ventilation or an open perforation into the pleural cavity, or progressive subcutaneous or mediastinal emphysema. Although there was an open perforation into the right pleural cavity in this case, it resolved with conservative treatment with stent placement. Therefore, surgical intervention is not necessarily indicated for all open perforations into the pleural cavity.

In conclusion, conservative treatment with a silicon Y-stent for ITI due to rigid bronchoscopy is safe in patients with uncomplicated ventilation or nonprogressive emphysema.

## Data Availability

All data supporting the findings of this study are included within the paper.
